# Chest X-ray findings monitoring COVID-19 disease course and severity

**DOI:** 10.1186/s43055-020-00296-x

**Published:** 2020-09-22

**Authors:** Rabab Yasin, Walaa Gouda

**Affiliations:** 1 Faculty of Medicine, Menofia University, Menofia, Egypt; 2 Radiology Department, Menofia University, Menofia, Egypt

**Keywords:** COVID-19, Coronavirus infections, X-ray, Pneumonia, Viral, Lung diseases

## Abstract

**Background:**

Coronavirus related respiratory illness usually manifests clinically as pneumonia with predominant imaging findings of an atypical or organizing pneumonia. Plain radiography is very helpful for COVID-19 disease assessment and follow-up. It gives an accurate insight into the disease course.

We aimed to determine the COVID-19 disease course and severity using chest X-ray (CXR) scoring system and correlate these with patients’ age, sex, and outcome.

**Results:**

In our study, there were 350 patients proven with positive COVID-19 disease; 220 patients (62.9%) had abnormal baseline CXR and 130 patients (37.1%) had normal baseline CXR. During follow-up chest X-ray studies, 48 patients (13.7%) of the normal baseline CXR showed CXR abnormalities. In abnormal chest X-ray, consolidation opacities were the most common finding seen in 218 patients (81.3%), followed by reticular interstitial thickening seen in 107 patients (39.9%) and GGO seen in 87 patients (32.5%). Pulmonary nodules were found 25 patients (9.3%) and pleural effusion was seen in 20 patients (7.5%). Most of the patients showed bilateral lung affection (181 patients, 67.5%) with peripheral distribution (156 patients, 58.2%) and lower zone affection (196 patients, 73.1%). The total severity score was estimated in the baseline and follow-up CXR and it was ranged from 0 to 8. The outcome of COVID-19 disease was significantly related to the age, sex, and TSS of the patients. Male patients showed significantly higher mortality rate as compared to the female patients (*P* value 0.025). Also, the mortality rate was higher in patients older than 40 years especially with higher TSS.

**Conclusion:**

Radiographic findings are very good predictors for assessing the course of COVID-19 disease and it could be used as long-term consequences monitoring.

## Background

The pandemic related to the coronavirus is now considered one of the deadliest epidemics. The numbers of cases exponentially increased with no specific treatment or promising vaccine in sight, creating havoc for the health and financial systems of the world [1–6].

Wuhan, the capital city of Hubei in China, reported the earliest cases that were treated as an unusual pneumonia. With the disease progression, the World Health Organization (WHO) announced the presence of several similar cases [7, 8].

Researchers revealed that a novel strain of the family *Coronaviridae* is the pathogen responsible for the respiratory illness of this disease and this is simulating two previous epidemics, namely MERS (Middle Eastern respiratory syndrome) and SARS (severe acute respiratory syndrome). The disease induced by SARS-CoV-2 was labeled as COVID-19 by the International Classification of Diseases (ICD) [9].

Chest radiographs are usually of limited value in the diagnosis of early stages especially in mild disease course; however, the CT findings may be present early even before the onset of the symptoms. Chest radiographs is very helpful in the intermediate to advanced stages of COVID-19 with features of acute respiratory distress syndrome (ARDS) as well as the follow-up [10–17].

CT is now considered the main investigator for COVID-19 creating so much burden on the departments of radiodiagnosis and requiring intense infection control measurements [5–11]. Moreover, some hospitals dedicated specific CT scanners for scanning suspected COVID-19 patients, that is why the American College of Radiology finds this may disrupt the availability of radiological services and recommended portable chest X-ray (CXR) as a first-line triage tool [17–22].

For better assessment of the role radiography in COVID-19 management, our study aims to describe the chest X-ray findings of COVID-19 and correlate this to the disease severity in review of patients’ age and sex.

## Methods

A retrospective study was performed during the period of 1st of April to 10th of June 2020 for 350 patients who tested positive for novel coronavirus by nasopharyngeal swap. Age of the patients ranged from 12 to 81 years old with mean = 41.68 ± 14.12. There were 261 males and 89 females with male to female distribution of 2.9:1.

### Image acquisition and analysis

Using the usual local protocols, all CXRs were obtained as computed or digital radiographs. PA projection was performed for the cases at the initial CXR at the time of presentation. AP projection was done during follow-up cases in the isolation wards using portable X-ray units.

### Image analysis

**Fig. 1:**
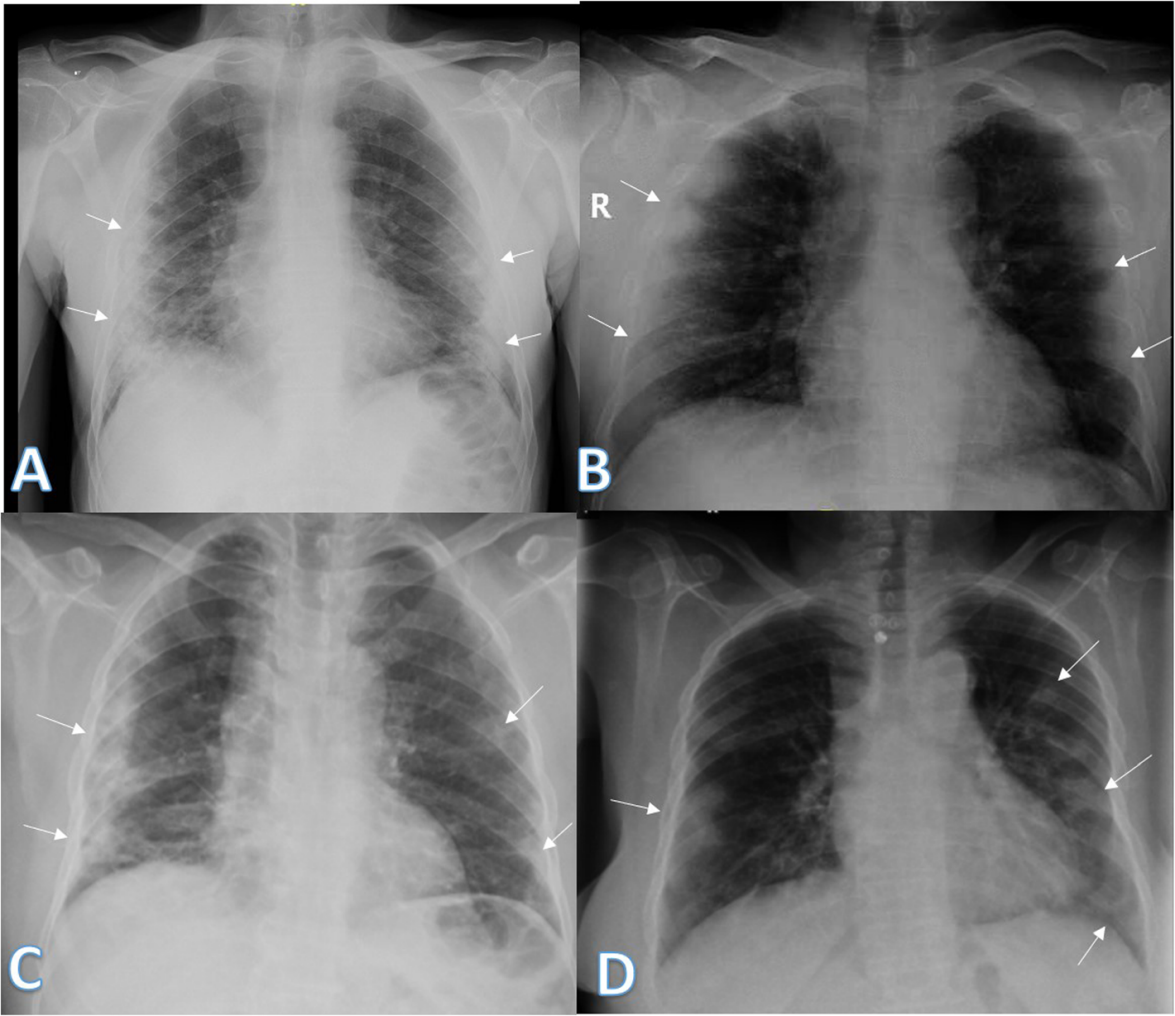
Four different patients with positive COVID-19. **a** 72-year male was complaining of fever and cough. Initial chest X-ray showed bilateral peripheral mid and lower zonal air space consolidation opacities (arrows). The severity score was 2 for each lung, so TSS was 4. **b** 44-year male was complaining of SOB, cough, and fever. Initial chest X-ray showed bilateral peripheral zonal air space consolidation opacities along the periphery of both lungs (arrows). The severity score was 2 for each lung so, TSS was 4. **c** 63-year male was complaining of cough and fever. Initial chest X-ray showed right peripheral mid and lower zonal air space consolidation opacities (arrows), smaller patches of consolidation is seen on the side (arrows), the severity score for the right lung was 2 and for the left lung was 1, so TSS was 3. **d** 68-year female patient was complaining of SOB and cough. Initial chest X-ray showed bilateral air space consolidation opacities (arrows) of peripheral midzonal distribution in the right lung and neither peripheral nor perihilar mid and lower zonal distribution in the left lung (arrows). The severity score for the right lung was 1 and for the left lung was 2, so the total severity score was 3

**Fig. 2:**
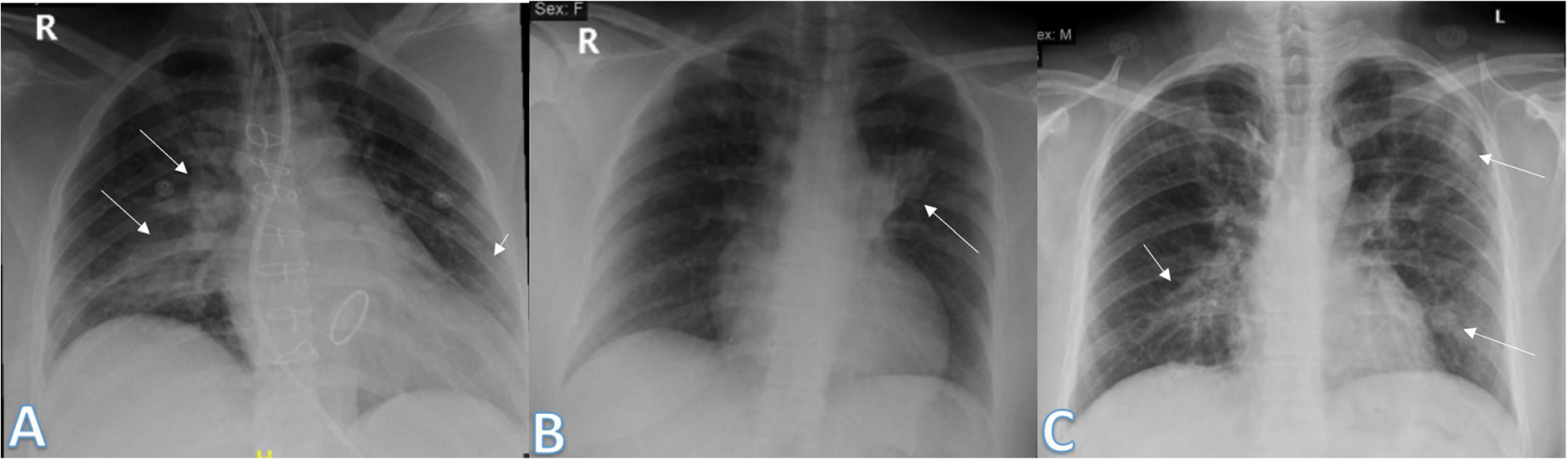
Three different patients with positive COVID-19. **a** 39-year female was complaining of SOB with tachypnea, fever, and cough. She had previous open cardiac surgery with valve replacement. She was intubated due to hypoxemia. Chest X-ray showed right perihilar air space consolidation opacity extending to the right paracardiac region (long arrows). Another left lower zonal air space consolidation opacity was seen (short arrow); the severity score was 2 for the right lung and 1 for the left lung, so TSS was 3. **b** 44-year female was complaining of fever. Initial chest X-ray showed left perihilar air space consolidation opacity (arrow). The total severity score was 1. **c** 35-year male was complaining of cough and fever. Initial chest X-ray showed right perihilar air space consolidation opacity (short arrow). Other left lower zonal air space consolidation opacities are seen (long arrows) with reticular thickening; the severity score was 1 for each lung, so TSS was 2

**Fig. 3:**
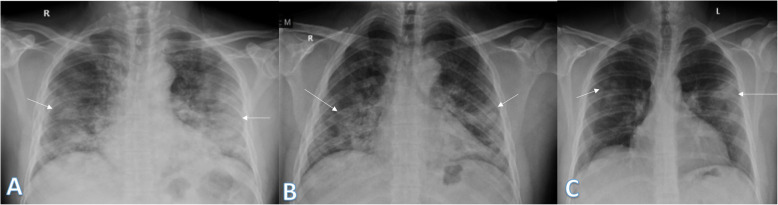
Three different patients with positive COVID-19. **a** 54-year male was complaining of SOB, fever, and cough. Initial chest X-ray showed bilateral mid and lower zonal air space consolidation opacities more on the left side with neither perihilar nor peripheral distribution (arrows). The severity score was 2 for the right lung and 3 for the left lung, so TSS was 5. **b** 36-year male was complaining of fever. Initial chest X-ray showed right perihilar (long arrow) and left neither peripheral nor perihilar air space consolidation opacity (short arrow) of the mid and lower lung zone. The severity score was 2 for each lung, so the total severity score was 4. **c** 27-year male was complaining of cough and fever. Initial chest X-ray showed right small midzonal pulmonary nodules (short arrow), left midzonal neither peripheral nor perihilar air space consolidation opacities are seen (long arrows), and the severity score was 1 for each lung, so TSS was 2

**Fig. 4:**
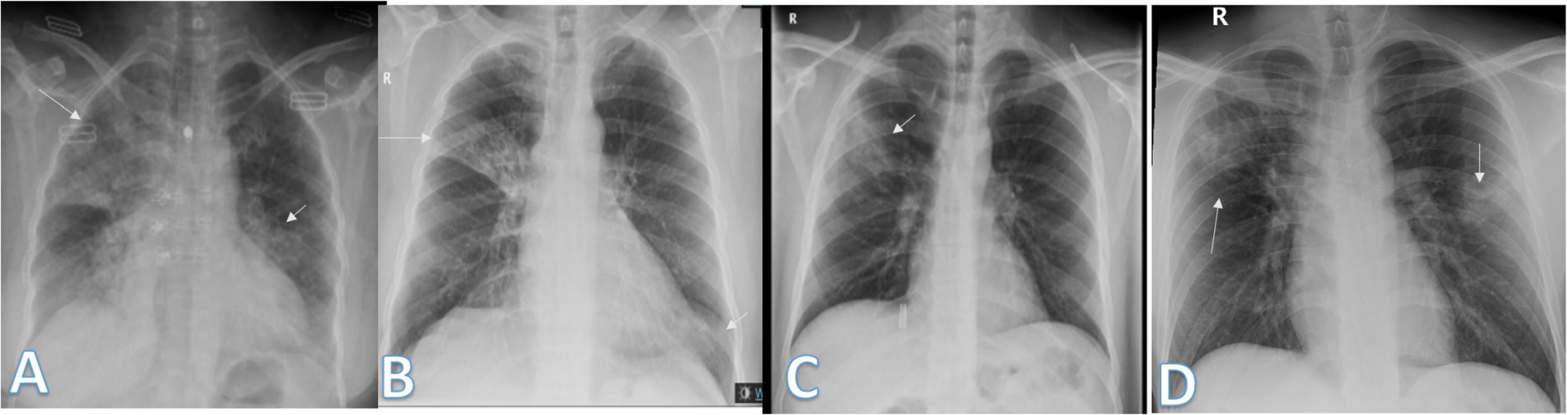
Four different patients with positive COVID-19. **a** 45-year female was complaining of fever and cough. Initial chest X-ray showed bilateral lower zonal air space consolidation opacities with upper zonal predominance on the right side (long arrow) and perihilar pattern on the left side. The severity score was 2 for the right lung and 1 on the left lung, so TSS was 3. **b** 60-year male was complaining of fever. Initial chest X-ray showed bilateral air space consolidation opacities with upper zonal predominance on the right side (long arrow) and at the paracardiac area on the left side (short arrow). The severity score was 1 for each lung so, TSS was 2. **c** 23-year asymptomatic male. Initial chest X-ray showed right upper zonal air space consolidation opacity (arrow). The total severity score was 1. **d** 28-year male patient presented with cough, on initial X-ray showed right upper zonal (long arrow) and left midzonal (short arrow) air space consolidation opacities; the severity score was 1 for each lung, so TSS was 2

**Fig. 5:**
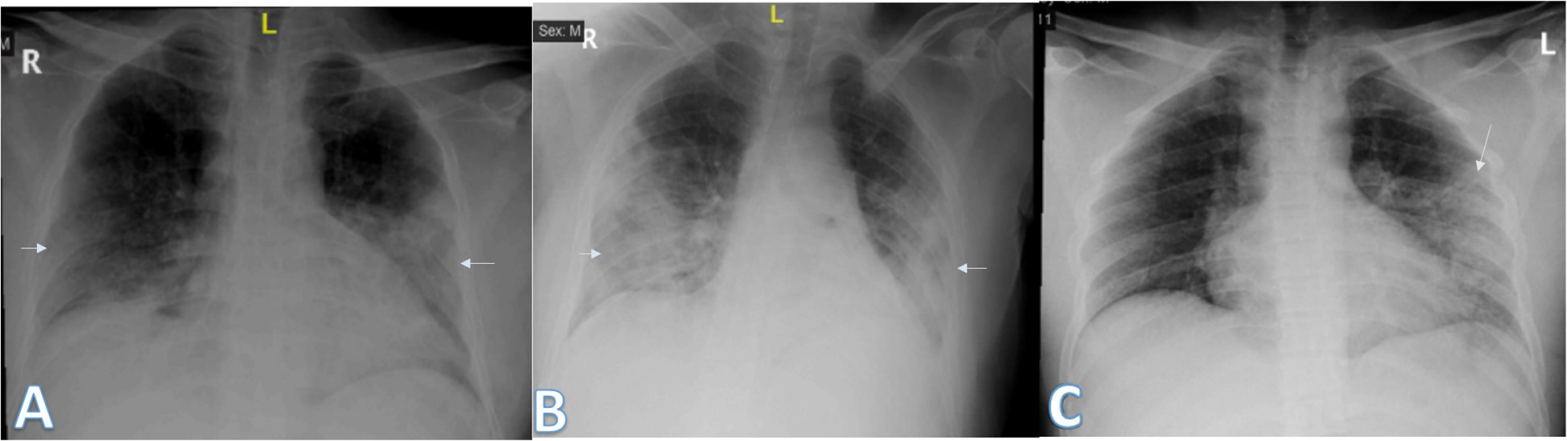
Three different patients with positive COVID-19. **a** 29-year male was complaining of SOB, fever, and cough. Initial chest X-ray shows bilateral lower zonal air space consolidation opacities (arrows). The severity score was 2 for each lung, so TSS was 4. **b** 52-year male was complaining of dyspnea and fever. Initial chest X-ray shows bilateral air space consolidation opacities (arrows) at both mid and lower lung zones (with lower zonal predominance). The severity score was 3 for each lung, so TSS was 6. **c** 32-year male patient was complaining of SOB and cough. Initial chest X-ray shows air space consolidation opacity (arrow) at the left mid and lower lung zones (with lower zonal predominance). The total severity score was 3

**Fig. 6:**
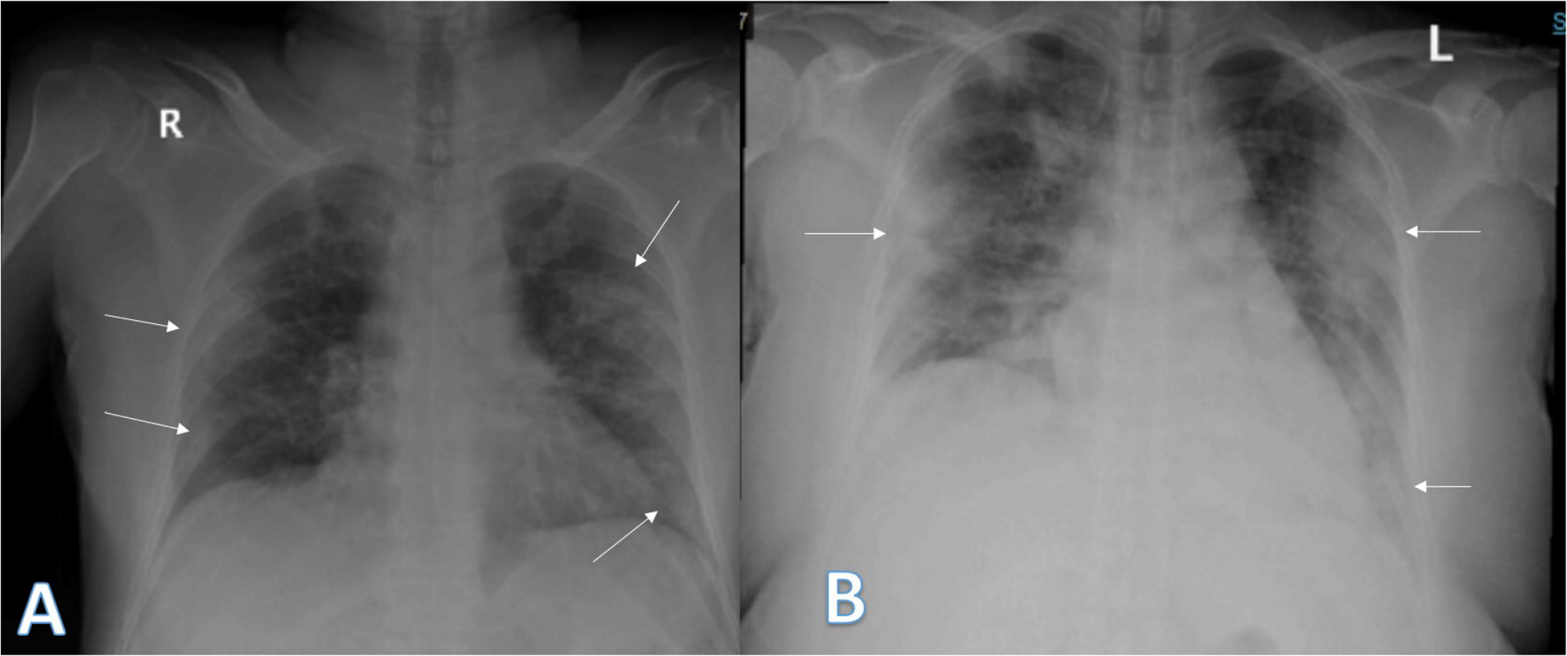
Two different patients with positive COVID-19. **a** 52-year male was complaining of fever, SOB, and cough. Initial chest X-ray showed right (peripheral) and left mid and lower zonal air space consolidation opacities with midzonal predominance (arrows). The severity score was 2 for the right lung and 3 on the left lung, so TSS was 5. **b** 36-year female was complaining of cough and fever. Initial chest X-ray showed mid and lower zonal air space consolidation opacities on both lungs with midzonal predominance (arrows). The severity score was 2 for the right lung and 3 on the left lung, so TSS was 5

Imaging findings were analyzed by 2 radiologists with experience of 20 years. The radiographic findings were classified as regards (i) peripheral predominance (Fig. 1), perihilar predominance (Fig. 2) (peripheral and perihilar demarcation was defined as halfway between lateral edge of the lung and hilum), or neither (Fig. 3); (ii) right, left, or bilateral lung affection; and (iii) upper (Fig. 4) and lower zonal (Fig. 5) or no zonal predominance (Fig. 6).

**Fig. 7:**
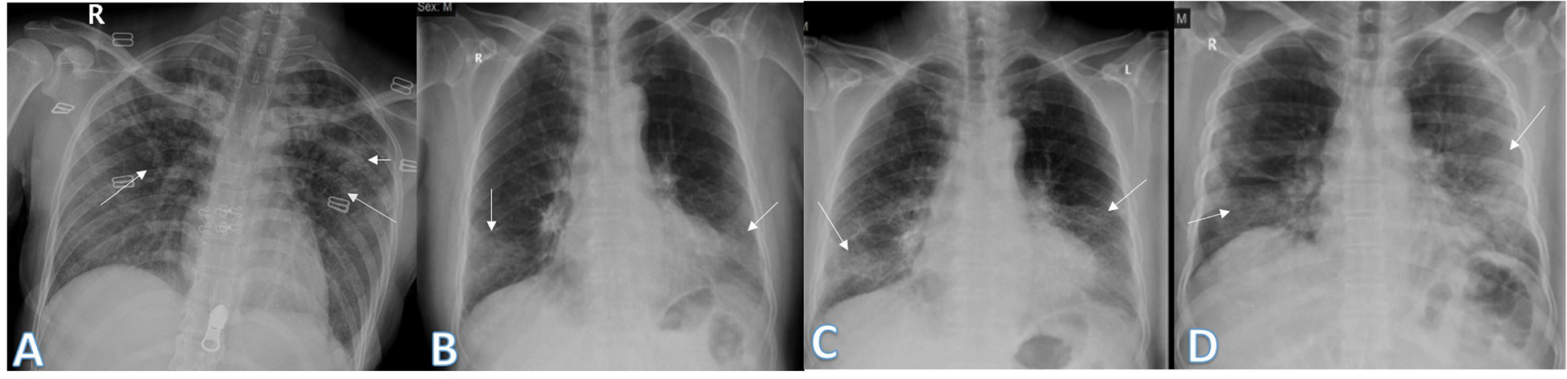
Four different patients with positive COVID-19. **a** 29-year female was complaining of cough and fever. Initial chest X-ray showed bilateral upper zonal ground glass opacities (long arrows) with small left upper zonal air space consolidation opacities (short arrow). The severity score was 2 for each lung so, TSS was 4. **b**, **c** 74-year male was complaining of dyspnea and fever. Initial and 1st follow-up chest X-ray showed bilateral lower zonal ground glass opacities (arrows). The total severity score was 3 in **b** and 4 in **c**. **d** 54-year male patient was complaining of SOB and cough. Initial chest X-ray show showed right lower zonal and left mid and lower zonal ground glass opacities (arrows). The severity score for the right lung was 1 and for the left lung was 3, so the total severity score was 4

**Fig. 8:**
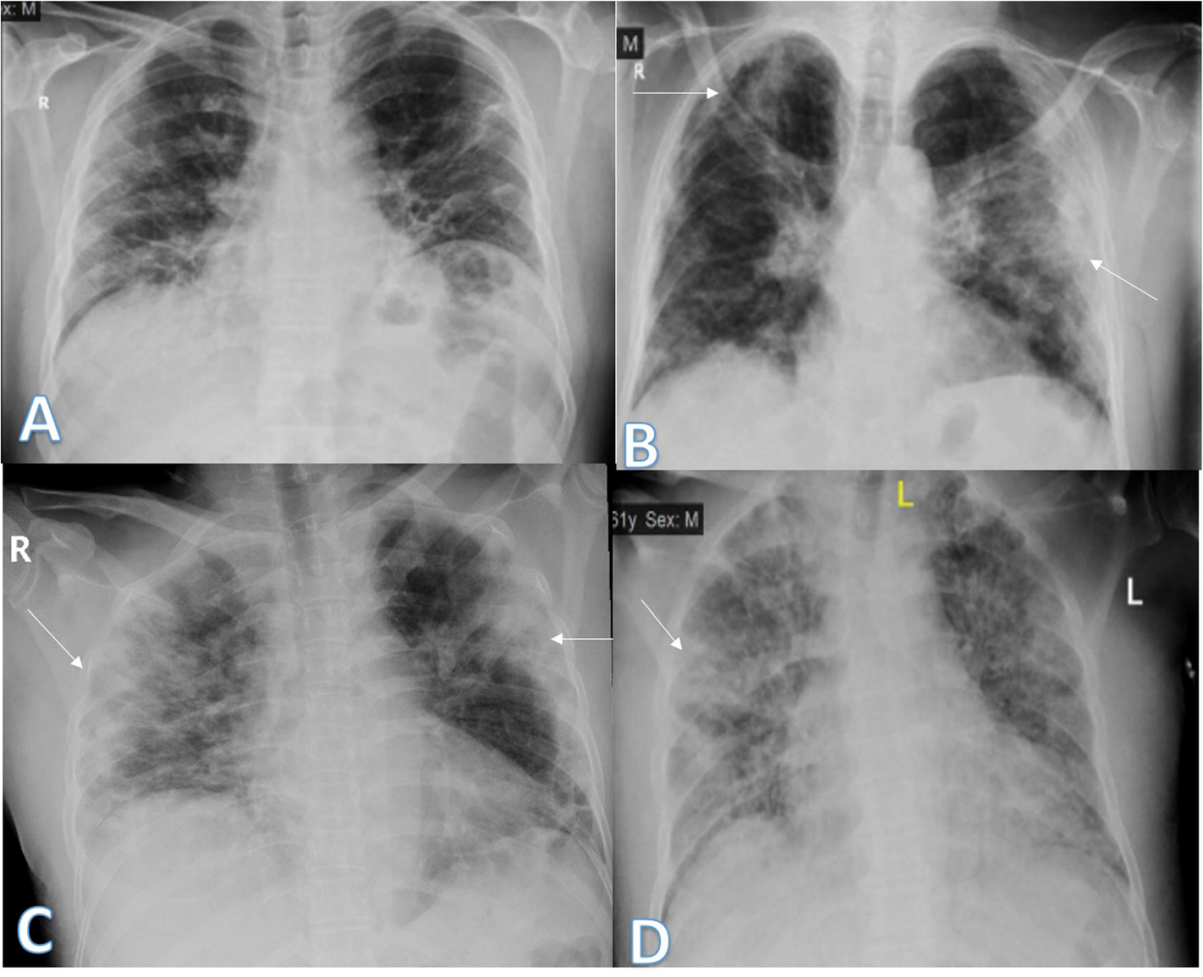
Four different patients with positive COVID-19. **a** 48-year male, **b** 48-year male, **c** 46-year male, and **d** 61-year male, follow-up chest X-ray showed bilateral diffuse reticular interstitial lung thickening with air space consolidation opacities were still seen (arrows)

**Fig. 9:**
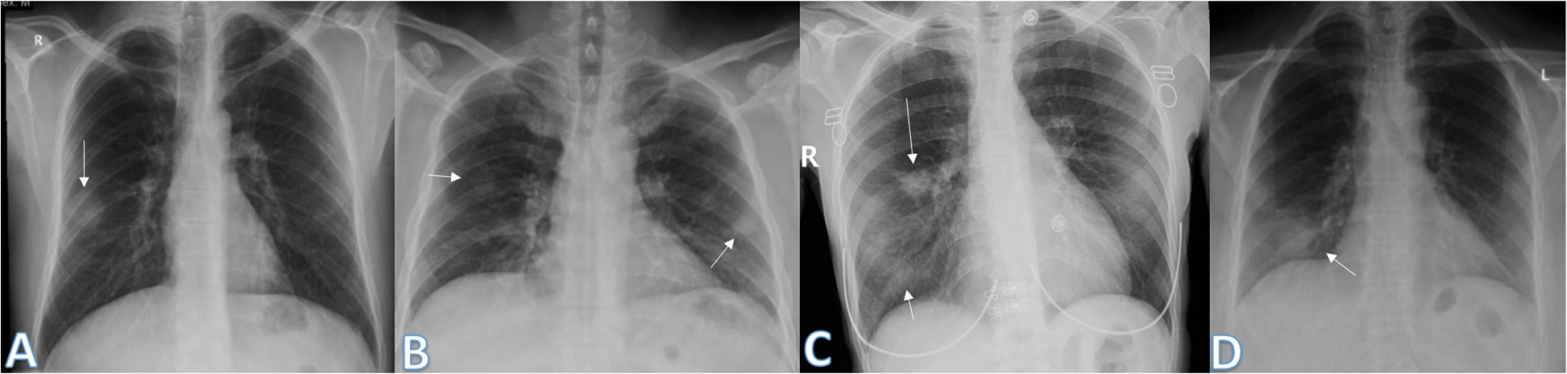
Four different patients with positive COVID-19. **a** 32-year male was complaining of cough. Initial chest X-ray showed a well-defined solitary pulmonary nodule at the lateral aspect of the right middle lung zone (arrow). The total severity score was 1. **b** 49-year male was complaining of fever. Initial chest X-ray showed two small well-defined pulmonary nodules at both middle lung zones (arrows). The severity score was 1 for each lung, so TSS was 2. **c** 36-year female was complaining of sore throat. Initial chest X-ray showed right perihilar fairly defined pulmonary nodule (long arrow) and right lower zonal ill-defined ground glass opacity (short arrow). The total severity score was 2. **d** 52-year female patient presented with cough, showed a well-defined solitary pulmonary nodule at the right lower lung zone (arrow). The total severity score was 1

**Fig. 10:**
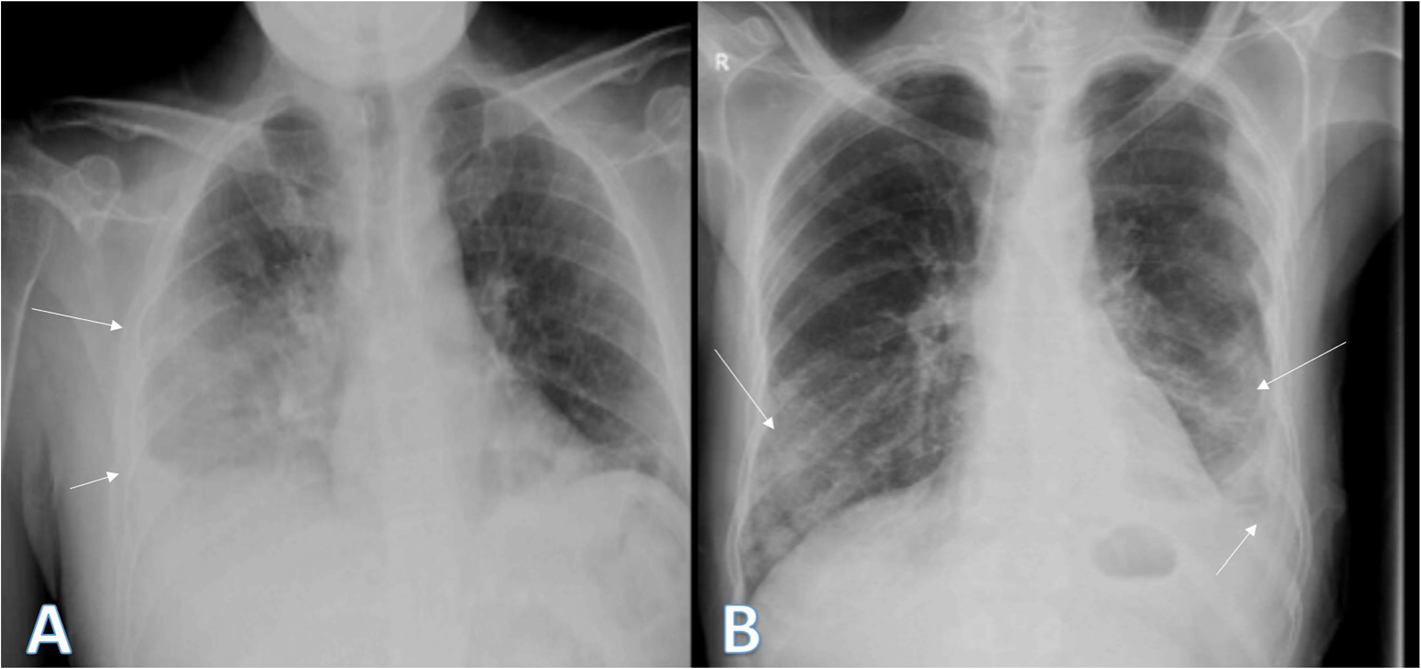
Two different patients with positive COVID-19. **a** 34-year male was complaining of cough and fever. Follow-up chest X-ray showed mild right pleural effusion (short arrow) with right mid and lower zonal air space consolidation opacities (long arrow). The total severity score was 2. **b** 61-year male was complaining of fever, cough, and diarrhea. Follow-up chest X-ray showed mild left pleural effusion with bilateral lower zonal air space consolidation opacities (long arrows). The severity score was 2 for each lung, so TSS was 4

The lung affection was assessed for the consolidation, ground glass opacities (GGO) (Fig. 7), reticulation interstitial thickening (Fig. 8), and pulmonary nodules (Fig. 9) as well as the presence of pleural effusion (Fig. 10) or pneumothorax.

### Radiographic scoring

Patients were classified according to the stage of illness into four groups (1–4 days, 5–9 days, 10–15 days, and > 15 days). The degree of disease severity was assessed using the severity score proposed by Warren et al [23]. Each lung was given a score of 0–4 depending on the extent of lung involvement (score 0 = no involvement; 1 ≤ 25%; 2 = 25–50%; 3 = 50–75%; 4 ≥ 75% lung affection). A total severity score was calculated by summing both lung scores (total severity scores ranged from 0 to 8). The patients were divided into four groups according to age: ˂ 20 years, 20–40 years, 40–59 and ≥ 60 years. Patients’ age, sex, and the highest CXR total severity score were correlated to the patients’ outcome.

### Statistical analysis of the collected data

Data were analyzed by an IBM compatible personal computer with SPSS statistical package version 23 (SPSS Inc. Released 2015. IBM SPSS statistics for windows, version 23.0, Armonk, NY: IBM Corp.), and were expressed as Number (No.), percentage (%), mean ($\overline{\mathrm{X}}$), and standard deviation (SD). Student’s *t* test was used for comparison of quantitative variables between two groups of normally distributed variables, while Mann Whitney’s test was used for not normally distributed ones. Chi-square test (*χ*^2^), with *Z* test to compare column proportions, was used to study association between qualitative variables but whenever any of the expected cells were less than five, Fischer’s exact test was used. Two-sided *P* value of < 0.05 was considered statistically significant.

## Results

This study was a retrospective one with the plain radiography done for 350 patients who tested positive for coronavirus by nasopharyngeal swap. Age of the patients ranged from 12 to 85 years old with mean age was 41.68 ± 14.12 years. There were 261 males and 89 females with male to female distribution of 2.9:1 (Table 1).

**Table 1:** Demographic presentations of the patients

Variable	No. (%)
**Age in years (mean ± SD, range)**	41.68 ± 14.12, 12.0–85.0
**Gender**	
Female	89 (25.4)
Male	261 (74.6)
**Outcome**	
Died	21 (6.0)
Alive	329 (94.0)

Baseline CXRs were performed for all patients at the time of initial presentation. Normal baseline CXRs were found in 130 patients (37.1%), while 220 patients (62.9%) had abnormal baseline CXR. During follow-up chest X-ray studies, 48 patients (13.7%) of the normal baseline CXR showed CXR abnormalities. So CXRs abnormalities were detected in 268 of 350 patients (77%) at certain points of the disease course (Table 2).

**Table 2:** The distribution and frequency of the radiographic findings.

	No. (%)
**X-ray findings**	
Negative	82 (23.4)
Initial negative then become positive	48 (13.7)
Positive from start	220 (62.9)
**Initial X-ray (*n*** **= 350)**	
Negative	130 (37.1)
Positive	220 (62.9)
**1st follow-up X-ray (1–4 days) (*n*** **= 276)**	
Negative	40 (14.5)
Positive	236 (85.5)
**2nd follow-up X-ray (5–9 days) (*n*** **= 167)**	
Negative	29 (17.4)
Positive	138 (82.6)
**3rd follow-up X-ray (10–15 days) (*n*** **= 135)**	
Negative	39 (28.9)
Positive	96 (71.1)
**4th follow-up X-ray (> 15 days) (*n*** **= 132)**	
Negative	51 (38.6)
Positive	81 (61.4)
**Affected lung**	
Right	42 (15.7)
Left	45 (16.8)
Both	181 (67.5)
**Distribution**	
Perihilar	31(11.6)
Peripheral	156 (58.2)
Neither	81 (30.2)
Ground glass appearance	87 (32.5)
Consolidation	218 (81.3)
Reticular thickening	107 (39.9)
Pulmonary nodules	25 (9.3)
Pleural effusion	20 (7.5)
**Predominance**	
Upper zone	17 (6.4)
Lower zone	196 (73.1)
Non-zonal	55 (20.5)
**Maximum severity score**	
0	82 (23.4)
1	57 (16.2)
2	91 (26.0)
3	38 (10.9)
4	24 (6.9)
5	20 (5.7)
6	16 (4.6)
7	20 (5.7)
8	2 (0.6)
**Time to maximum total severity score**	
Initial X-ray	113 (42.2)
1–4 days	92 (34.3)
5–9 days	58 (21.6)
10–15 days	5 (1.9)

The most common CXRs features detected in COVID-19 cases were consolidation seen in 218 patients (81.3%), followed by reticular interstitial thickening seen in 107 patients (39.9%) and GGO seen in 87 patients (32.5%). Few cases showed pulmonary nodules seen in 25 patients (9.3%) and pleural effusion seen in 20 patients (7.5%) (Table 2).

**Fig. 11:**
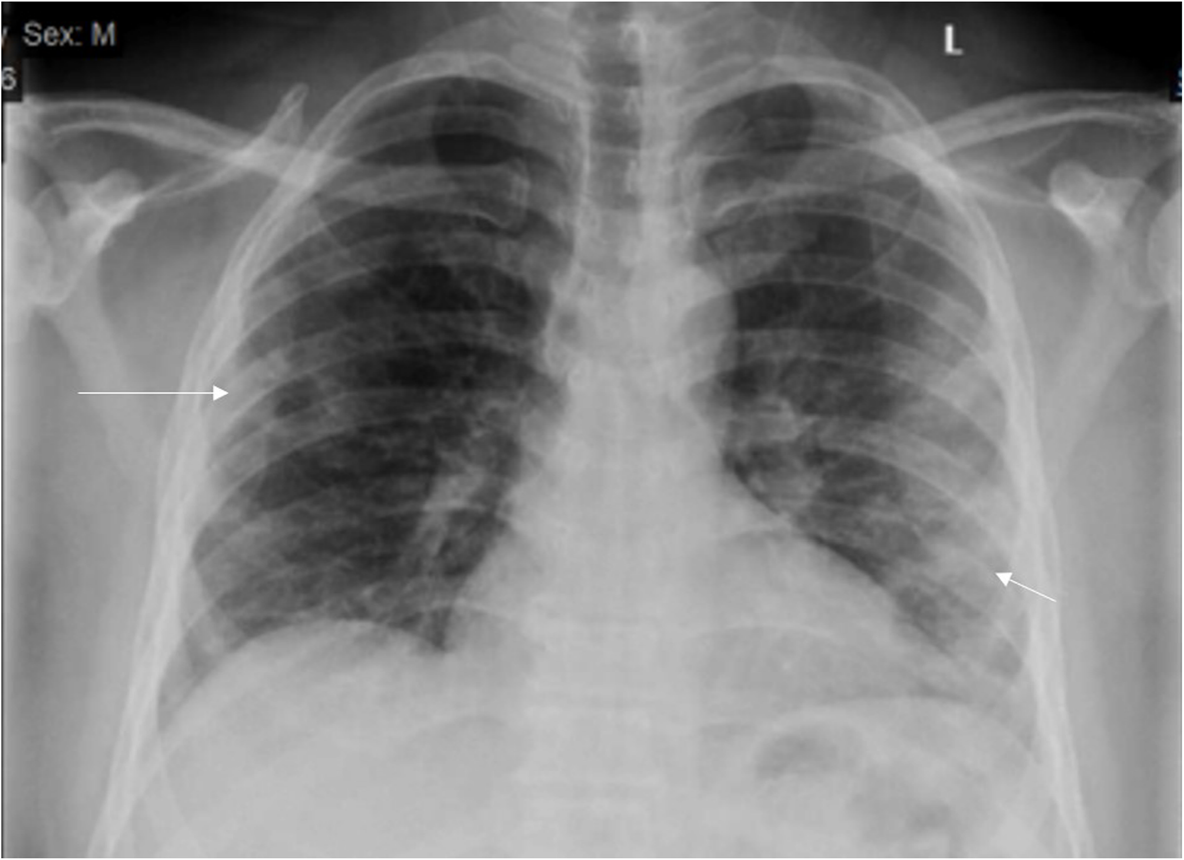
54-year male patient with positive COVID-19. He was complaining of cough and fever. Follow-up chest X-ray showed right midzonal lesion with central lucency giving atoll sign (long arrow) of organizing pneumonia. Also, left lower zonal small air space consolidation opacities with reticular thickening was seen (short arrow). The severity score was 1 for each lung, so the total severity score was 2

There were 2 patients with pneumothorax which was iatrogenic due to mechanical ventilation. One case of CXR shows Atoll sign (Fig. 11).

Most of the patients showed bilateral lung affection (181 patients, 67.5%), 42 patients (15.7%) had right and 45 patients (16.8%) had left lung affection (Table 2).

The zonal predominance of the lesions was classified as upper, lower, and non-zonal involvement. Most of the cases show lower zonal predominance (196, 73.1%), while 55 patients (20.5%) had non-zonal distribution and only 17 patients (6.4%) had upper zonal predominance. Most cases showed peripheral predominance (156 patients, 58.2%), while 31 patients (11.6%) had perihilar predominance (Table 2).

The total severity score was estimated in the baseline and follow-up CXR and it ranged from 0–8. Mild findings with total severity score ranging between 0 and 2 were found in 230 patients (65.7%) and moderate severity score ranging between 3 and 5 were found in 82 patients (23.4%), while severe cases with severity score ranging between 6 and 8 were found in 38 patients (10.9%) with more disseminated lung involvement (Table 2).

The time to reach the maximum total severity score was estimated (Table 2); 113 patients (42.2%) showed maximum TSS at the initial baseline CXR followed by 92 patients (34.3%) who showed maximum TSS at 1st follow-up CXR done (1–4 days).

Table 3 showed the severity score at different stages of the disease, the highest total severity score of the CXR findings was found in the 4th follow-up CXR 15 days after the onset of the symptoms with its mean 4.51 ± 1.61, while the lowest TSS was found in the initial baseline CXR with its 1.49 ± 1.53. The severity score 5–7 was found mostly along the 2nd and 3rd stages.

**Table 3:** CXRs severity score at different stages

Severity score	Initial X-ray (***n*** = 350)	1st follow-up X-ray (1–4 days) (***n*** = 274)	2nd follow-up X-ray (5–9) (***n*** = 166)	3rd follow-up X-ray (10–15) (***n*** = 140)	4th follow-up X-ray (> 15 days) (***n*** = 135)
	No. (%)	No. (%)	No. (%)	No. (%)	No. (%)
0	130 (37.1)	40 (14.6)	29 (17.5)	39 (27.9)	53 (39.3)
1	65 (18.6)	49 (17.9)	17 (10.2)	13 (9.3)	21 (15.6)
2	92 (26.3)	76 (27.7)	28 (16.9)	27 (19.3)	26 (19.3)
3	31 (8.9)	44 (16.1)	23 (13.9)	14 (10.0)	9 (6.7)
4	20 (5.7)	28 (10.2)	15 (9.0)	20 (14.3)	12 (8.9)
5	7 (2.0)	15 (5.5)	20 (12.0)	8 (5.7)	2 (1.5)
6	4 (1.1)	18 (6.6)	16 (9.6)	9 (6.4)	9 (6.7)
7	1 (0.3)	4 (11.5)	17 (10.2)	8 (5.7)	3 (2.2)
8	-	-	1 (0.6)	2 (1.4)	-
**Mean ± SD**	1.49 ± 1.53	2.08 ± 1.83	3.42 ± 2.32	3.60 ± 2.23	4.51 ± 1.61

**Fig. 12:**
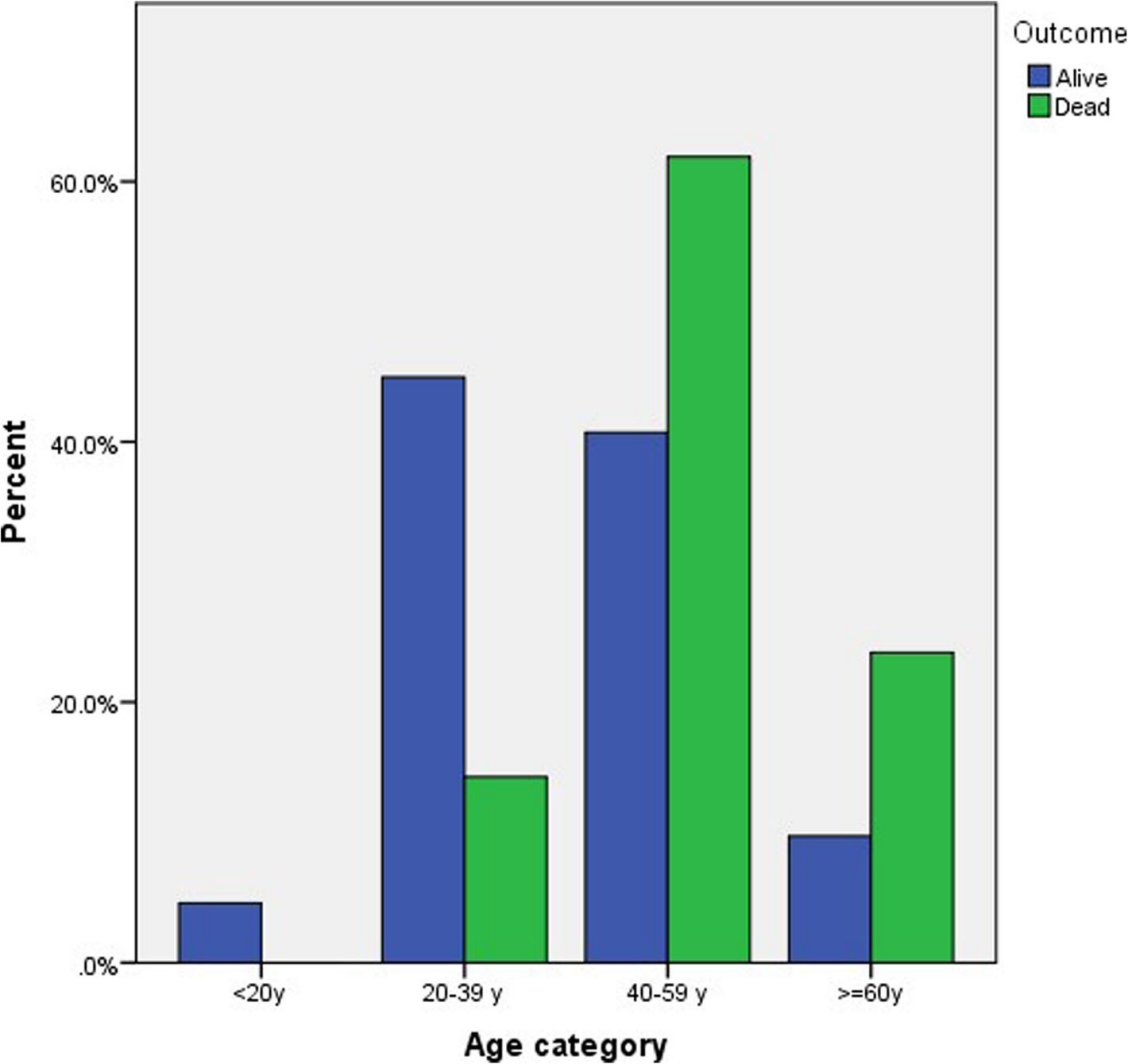
Percentage distribution of outcome according to age category

The patient’s outcome (alive or dead) was correlated to different variables as shown in Table 4. The mean age for the alive patients was 41.09 ± 14.14, while the mean age for the dead patients was 51.04 ± 10.17. The age of the patients was classified to 4 groups (˂ 20 years, 20–40 years, 40–59 and ≥ 60 years) with a significant difference between the age of the patients and COVID-19 disease outcome (*P* value = 0.008) (Fig. 12). Lowest mortality rate was observed in 20–40 years while patients aging 40–59 and ≥ 60 years shows significantly higher mortality rate.

**Table 4:** the correlation between the patient’s outcome to the age, sex and maximum severity score

Variables	Outcome		***P*** value
	Alive	Dead	
	Mean ± SD	Mean ± SD	
**Age**	41.09 ± 14.14	51.04 ± 10.17	0.002
**Age category**			
< 20 years	15 (4.6%)	0 (0.0)	0.008
20–39	148 (45.0%)*	3 (14.3%)	
40–59	134 (40.7%)	13 (61.9%) *	
60 or more	32 (9.7%)	5 (23.8%) *	
**Gender**			
Male	241 (73.3)	20 (95.2)	0.025
Female	88 (26.7)	1 (4.8)	
**Maximum total severity score**	2.06 ± 1.84	6.87 ± 0.71	< 0.001
**Maximum TSS**			
**0**	82 (24.6%)	0 (0.0%)	0.049
**1**	57 (17.1%)	0 (0.0%)	0.143
**2**	91 (27.2%)	0 (0.0%)	0.032
**3**	38 (11.4%)	0 (0.0%)	0.307
**4**	24 (7.2%)	0 (0.0%)	0.544
**5**	19 (5.7%)	1 (6.3%)	0.652
**6**	14 (4.2%)	2 (12.5%)	0.756
**7**	9 (2.7%)	11 (68.8%)	< 0.001
**8**	0 (0.0%)	2 (12.5%)	< 0.001
**Time to maximum score**			
Initial X-ray	113 (44.8%)	0 (0.0%)	0.001
1–4 days	91 (36.1%)	1 (6.3%)	0.030
5–9 days	45 (17.9%)	13 (81.3%)	< 0.001
10–15 days	3 (1.2%)	2 (12.5%)	0.022

*Significantly higher than their corresponding category in the other group (< 0.05)

Male patients showed significantly higher mortality rate compared to the female patients (*P* value 0.025). The disease outcome showed a positive correlation with the maximum severity score (6.87 ± 0.71 for the dead patients and 2.06 ± 1.84 for the survived patients) with high statistical significance (*P* value < 0.001) as well as to the time to reach this score (Table 4).

**Fig. 13:**
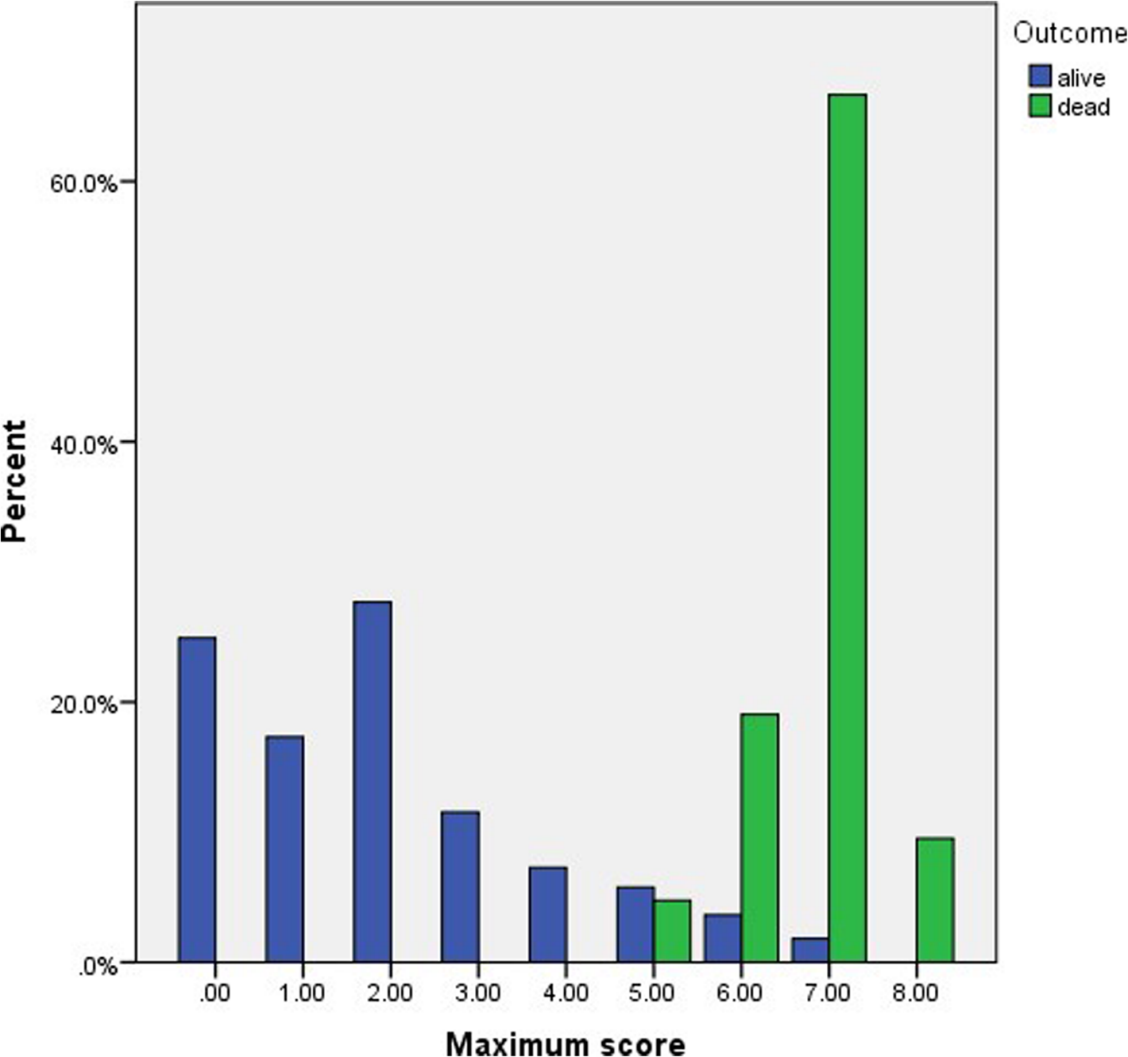
Percentage distribution of maximum score and outcome

In patients with TSS 2, there was a statistical significance between the TSS and the outcome of COVID disease for the survived patients (*P* value 0.032), while in patients with TSS 7 and 8, there was a highly statistical significance for the outcome for the dead patients (*P* value < 0.001) (Fig. 13).

**Fig. 14:**
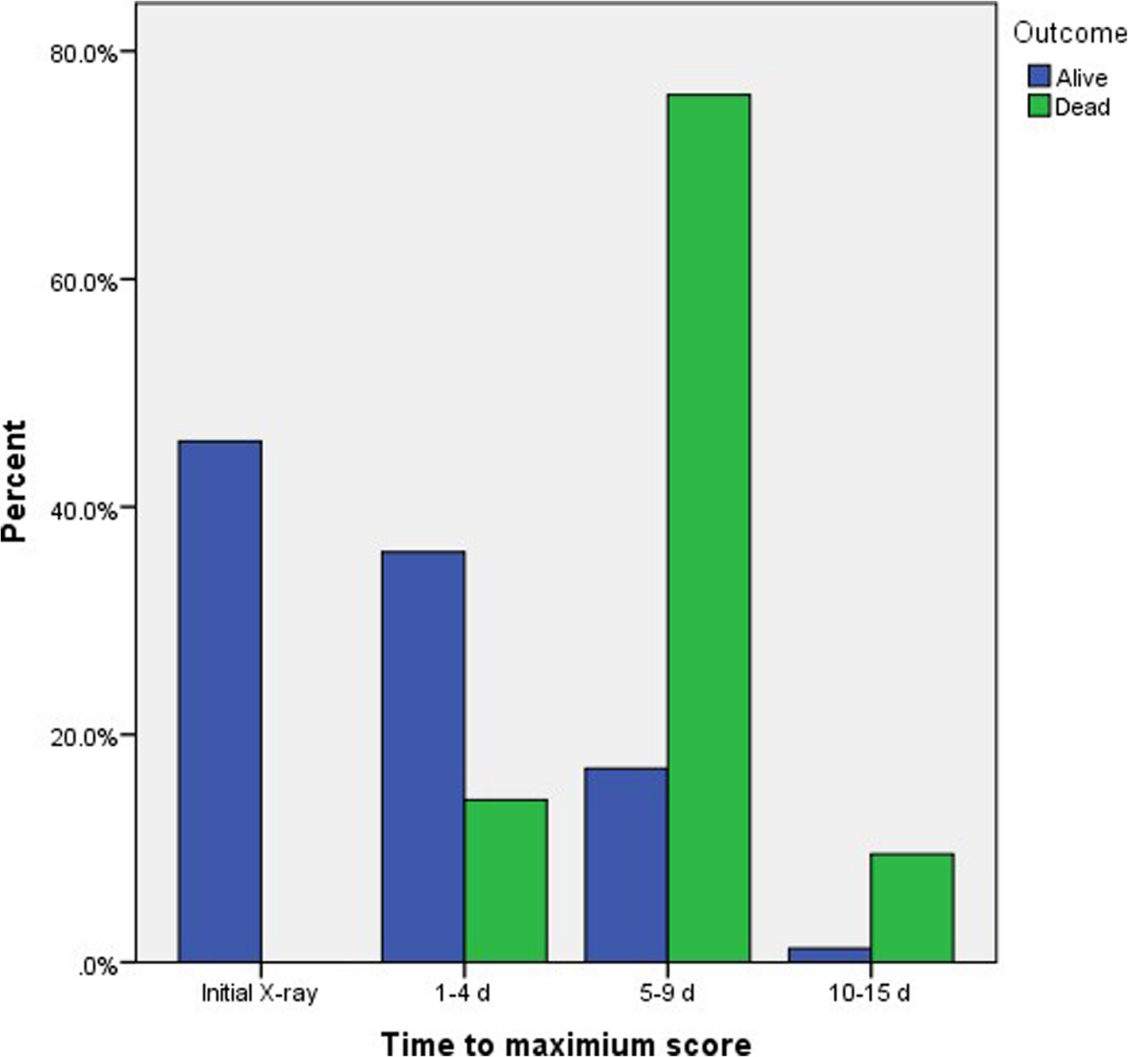
Percentage distribution of time to maximum score and outcome

In our study, there was a statistical significance between the time to reach the maximum TSS and outcome of COVID infection (Fig. 14). In initial chest X-ray and 1st follow-up chest X-ray (1–4 days), there was statistically significant for the survived group (*P* value 0.001 and 0.03, respectively). In 2nd and 3rd follow-up chest X-ray (5–15 days), there was a statistical significance for the dead group (*P* value < 0.001 and 0.022, respectively).

## Discussion

COVID-19 is a highly infectious disease that has been spread widely through the world. The disease management strategies primarily depend upon the early disease diagnosis [3, 15, 16]. However, the dramatic dissemination of the disease created a great challenge due to the insufficient laboratory kits [17]. That is why radiology has become a forefront method during the outbreak of COVID-19 [18].

Current literatures are mostly assessing COVID-19 CT findings, as it offers more sensitive results than chest X-ray (CXR) especially in the initial assessment of the patients.

The increased number of hospitalized patients and the consequent increase in radiological examinations would make the constant use of chest CT scan (from diagnosis to discharge) difficult to sustain over time [24]. The dependence on CT creates a huge burden on radiology departments and this makes the CXRs greatly substitute the CT examinations [1–4].

Although chest X-ray (CXR) is considered less sensitive for the detection of pulmonary involvement in early-stage disease, it is useful for monitoring the rapid progression of lung abnormalities in COVID-19, especially in critical patients admitted to intensive care units [24].

To provide valuable help for the clinicians and improve the stratification of the disease risk, chest X-ray (CXR) scoring system was tailored providing a semi-quantitative tool for assessment of lung abnormalities [5].

In this study, we analyzed the CXRs findings and severity scores of patients proven to have COVID-19 in different stages of disease. CXRs abnormalities were detected in 268 of 350 patients (77%) at certain points of the disease course.

In our study, each lung was given a score of 0–4 depending on the extent of lung involvement (score 0 = no involvement; 1 ≤ 25%; 2 = 25–50%; 3 = 50–75%; 4 ≥ 75% lung affection). A total severity score was calculated by summing both lung scores (total severity scores ranged from 0 to 8).

Borghesi et al. made another CXR scoring system for COVID-19 pneumonia (Brixia score) by dividing the lungs to six zones on frontal projection (upper, middle, and lower zones); then, a score (from 0 to 3) is assigned to each zone based on the lung abnormalities detected on frontal chest projection as follows: score 0, no lung abnormalities; score 1, interstitial infiltrates; score 2, interstitial and alveolar infiltrates (interstitial predominance); and score 3, interstitial and alveolar infiltrates (alveolar predominance). The scores of the six lung zones are then added to obtain an overall “CXR SCORE” ranging from 0 to 18 [24].

**Fig. 15:**
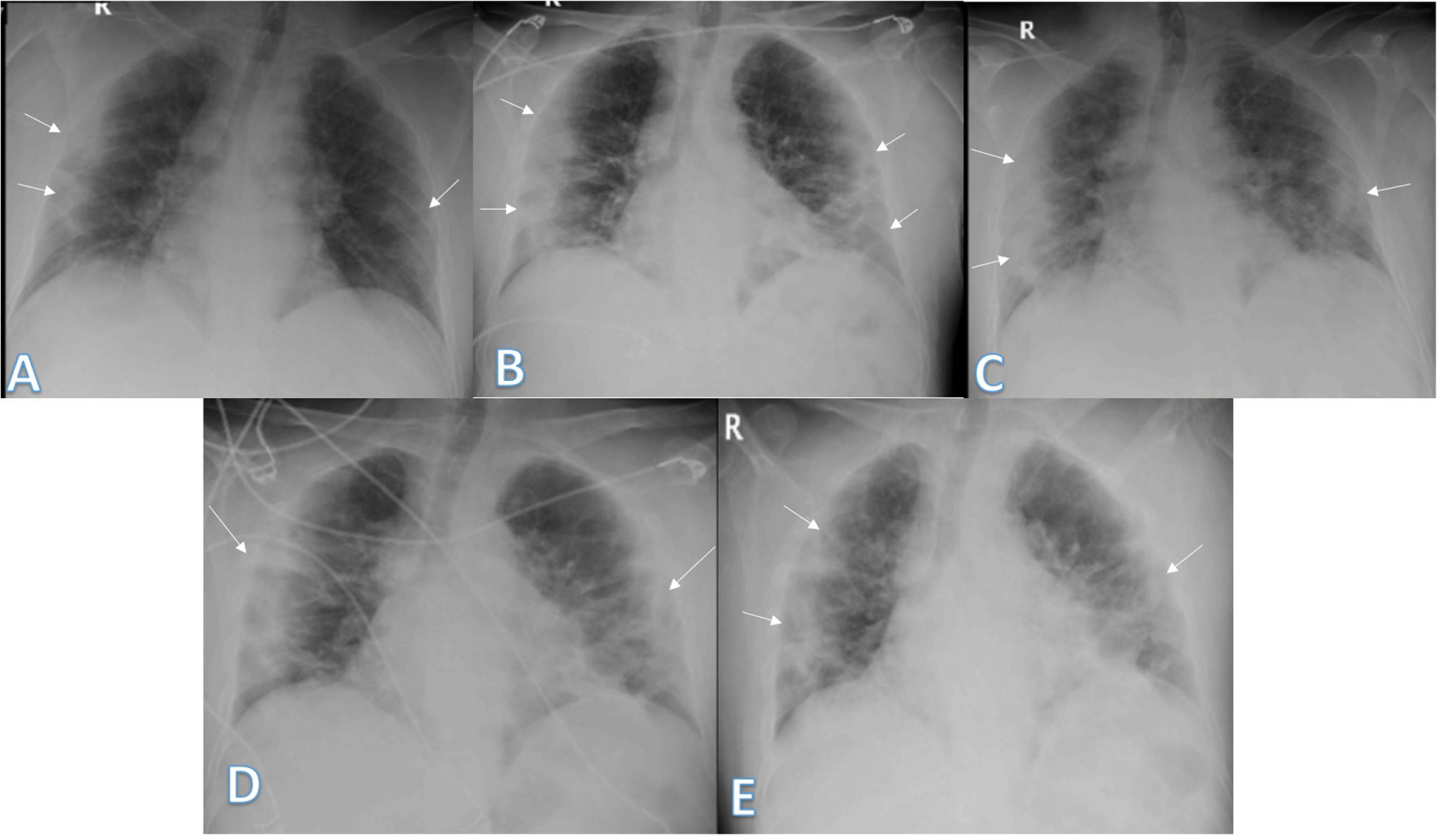
52-year male with positive COVID-19 virus. Initial chest X-ray (**a**) showed air space consolidation opacities at the periphery of the mid and lower zones of the right lung and small patch at the left lower lung zone (arrows) with TSS 3 (2 on the right side and 1 on the left side). On the 3rd day (**b**), air space consolidation opacities (arrows) increased on the left side and TSS became 4. On the 5th day (**c**), 8th day (**d**)**,** and 12th day (**e**), air space consolidation opacities (arrows) were almost the same but with increased reticular interstitial thickening seen on **d** and **e**

**Fig. 16:**
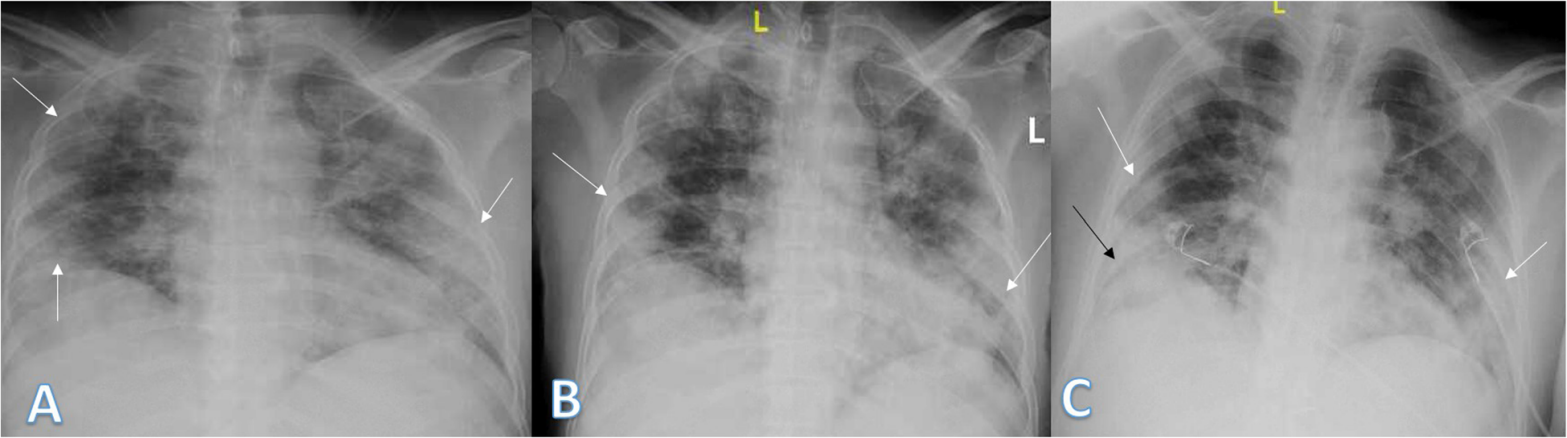
48-year male with positive COVID-19 virus. Initial chest X-ray (**a**) showed air space consolidation opacities at the periphery of the right lung and the mid and lower zones (arrows) with TSS as 4 (2 for each lung). On the 3rd day (**b**) and 6th day (**c**), air space consolidation opacities (arrows) were almost the same with mild right pleural effusion (black arrow on **c**)

In our study, most of the patients showed bilateral lung affection (181 patients, 67.5%) with lower zonal predominance (196, 73.1%) and peripheral distribution (156 patients, 58.2%) (Figs. 15 and 16).

The most common CXRs features detected in COVID-19 cases were consolidation seen in 218 patients (81.3%), followed by reticular interstitial thickening seen in 107 patients (39.9%) and GGO seen in 87 patients (32.5%). Few cases showed pulmonary nodules seen in 25 patients (9.3%) and pleural effusion seen in 20 patients (7.5%).

This agreed with Wong et al. who did a study on 64 COVID-19 patients, they found that Consolidation was the most common finding (47%), followed by GGO (33%). Also, peripheral predominance was seen in 41% of CXR abnormalities with lower zone distribution (50%) with bilateral lung involvement (50%). Pleural effusion was uncommon, only seen in 3% [5, 6].

Also, Lomoro et al. performed a study on thirty-two patients of COVID-19 disease; they found that consolidation is the most common finding (46.9%) with bilateral lung affection in (78.1%) and lower zone involvement (52%). No pleural effusion was identified [25].

Jacobi et al. stated that standard CXR can easily identify reticular opacities accompanying regions of ground glass attenuation. They state that air space consolidation opacities with peripheral and lower zone distribution are unique for COVID-19 disease [26].

Chen et al. reported bilateral pneumonia as the most common finding on chest radiograph [27]. While, Ng et al. reported that CXR lacks sensitivity in the early stages of lung disease [6].

In most studies, pleural effusions, pneumothorax, and lung cavitation are rare in COVID-19 infected patients [6, 26].

**Fig. 17:**
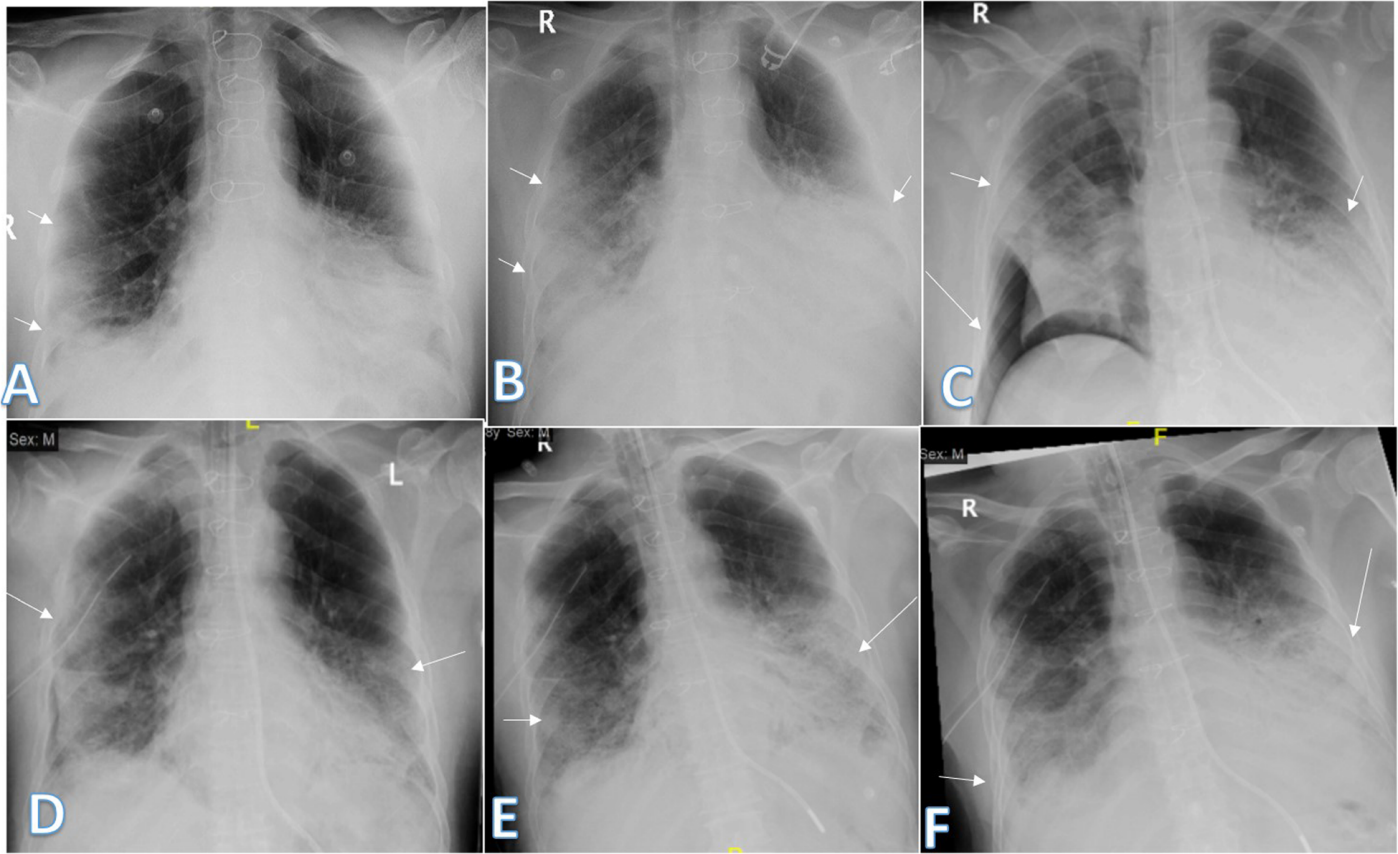
68-year male was complaining of SOB and high fever. He had done previous CABD. Initial chest-ray (**a**) showed peripheral air space consolidation at the right mid and lower lung zones (arrows) with TSS 2, 4 days later (**b**), air space consolidation opacities increased on the right side and appeared at the lower zone of left lung with TSS 5 (3 on RT and 2 on LT). The patient deteriorated and was intubated. On the 6th day (**c**), he developed right side pneumothorax (long arrow) and ICT was inserted. Bilateral air space consolidation opacities (small arrows) of total severity score 5. On the 11th day (**d**), pneumothorax decreased with TSS still 5. On the 16th day (**e**), he developed mild left side pleural effusion (long arrow) with bilateral consolidation opacities of TSS 6. On the 23rd (**f**), he developed mild right pleural effusion (arrow) with TSS 6. The patient died 1 day later

**Fig. 18:**
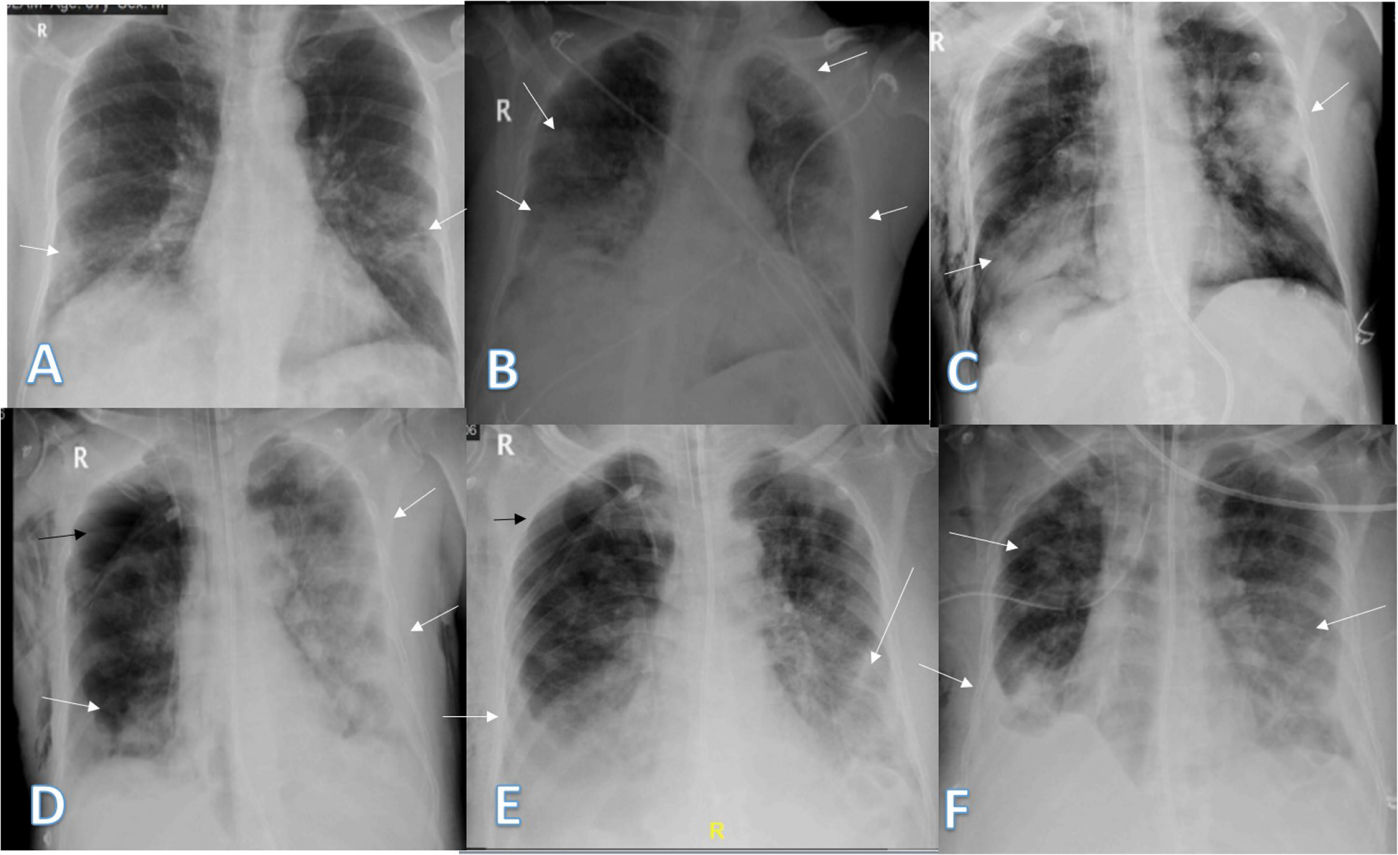
67-year male with positive COVID-19 virus. Initial X-ray (**a**) showed small right and left lower zonal midzonal air space consolidation opacities (arrows) with TSS as 2. On the 3rd day (**b**), air space consolidation opacities increased extended to involve most of the left lung and the lower zone of the right lung with midzonal patches (arrows); TSS reached 5 (3 on LT side and 2 on right side). The patient deteriorated and was hypoxemic, so he was intubated. On the 7th day (**c**), the patient developed pneumothorax and ICT was inserted. On the 9th day (**d**), 13th day (**e**), and 16th day (**f**), there was air space consolidation (arrows) more on the left lung with mild pneumothorax (black arrows on **d** and **e**). Minimal right pleural effusion was seen on (short arrows on **e** and **f**). The total severity score was 6 on **d**–**f**; the patient died

Pneumothorax was detected in 2 cases in our study and it was iatrogenic due to mechanical ventilation in intubated patients (Figs. 17 and 18).

**Fig. 19:**
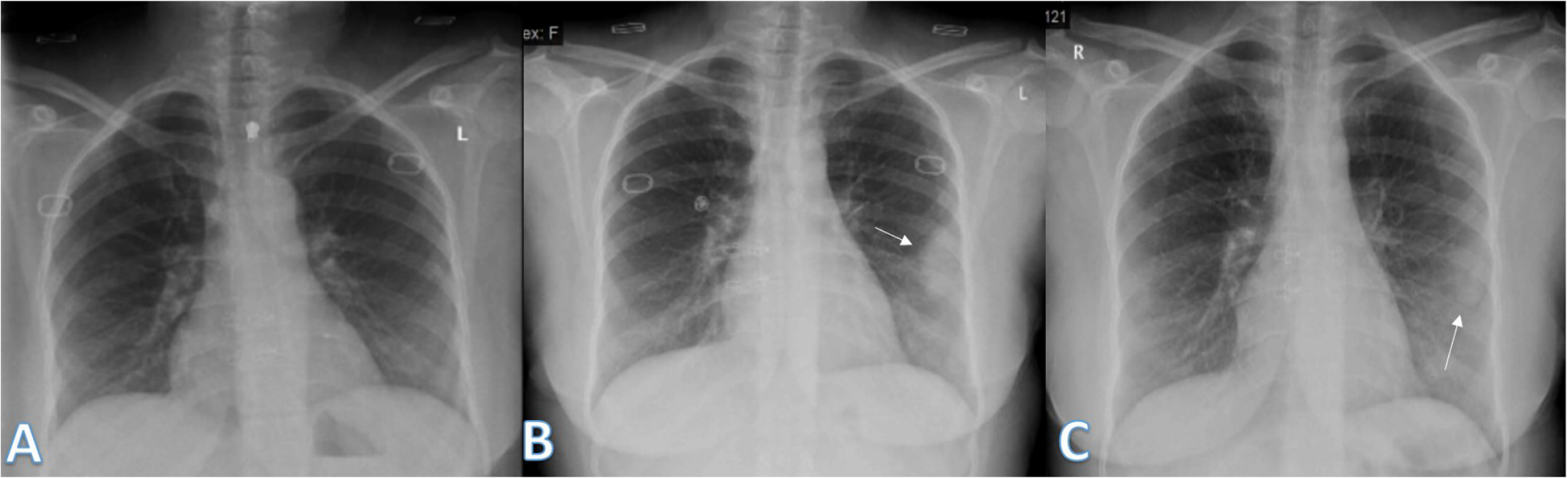
41-year female with positive COVID-19 virus. Initial chest X-ray (**a**) was normal. On the 3rd day, follow-up chest X-ray (**b**) shows a well-defined large solitary pulmonary nodule (arrow) at the periphery of the left middle lung zone with TSS as 1. On the 18th day (**c**), the lesion was partially resolved with faint density (arrow)

In our study, there was a case of large solitary pulmonary nodule which resolved on follow-up (Fig. 19). It had been reported by Jacobi et al. of large pulmonary nodule in COVID-19 patient [26].

We classified patients according to the stage of illness into four stages (1–4 days, 5–9 days, 10–15 days, and > 15 days). The degree of disease severity was assessed using semi-quantitative CXRs severity score that reflects the severity of different stages of this disease. The total severity score was lowest at stage 1 compared to other stages, with significant difference among other stages, indicating that the disease changed rapidly within 10–15 days after the onset of the initial symptoms.

We estimated the total severity score in the baseline and follow-up CXR, and it ranged from 0 to 8. In most cases (230 patients, 65.7%), TSS was mild, ranging between 0 and 2. While, in 82 patients (23.4%), there was moderate severity score ranging between 3 and 5. Severe cases with severity score of ranged between 6 and 8 was found in 38 patients (10.9%) with more disseminated lung involvement.

Wong et al. found in their study that 41% had mild findings with total severity score of 1–2, while moderate and severe cases with more extensive lung involvement was seen in 20% and 8% patients, who had severity scores of 3–4 and 5–6, respectively. There was no patient who had a severity score of > 6 on their baseline CXR with the severity of CXR findings peaked at 10–15 days from the date of symptom onset [5].

In our study, the maximum total severity score was reached in 113 patients (42.2%) in the initial baseline CXR with mean total severity score 1.49 ± 1.53 followed by 92 patients (34.3%) who reached the maximum TSS at 1st follow-up CXR done (done 1–4 days) with mean total severity score 2.08 ± 1.83.

The highest total severity score of the CXR findings was found in the 4th follow-up CXR 15 days after the onset of the symptoms with its mean 4.51 ± 1.61.

Our study correlated the disease outcome to the patients’ age with a significant difference between the age of the patients and COVID-19 disease outcome (*P* value = 0.008). The mean age for the recovered patients was 41.09 ± 14.14 while, the mean age for the dead patients was 51.04 ± 10.17. Lowest mortality rate was observed in 20–40 years, while patients aging 40–59 and ≥ 60 years showed significantly higher mortality rate.

In our study, there were 261 males (74.6%) and 89 females (25.4%) with male patients showing significantly higher mortality rate compared to the female patients (*P* value 0.025).

This agreed with Borghesi et al., who did a study on 783 Italian patients. They found that most patients (67.9%) were males and only 15.2% were younger than 50 years. They stated that in older age groups between 50 and 79 years, there was more significant pulmonary affection with highest severity score seen in males ≥ 50 years or female ≥ 80 especially that underlying comorbidities (such as hypertension, diabetes, cardiovascular disease, and oncologic history) are risk factors of fatal outcome in adult patients with confirmed SARS-CoV-2 infection [9].

**Fig. 20:**
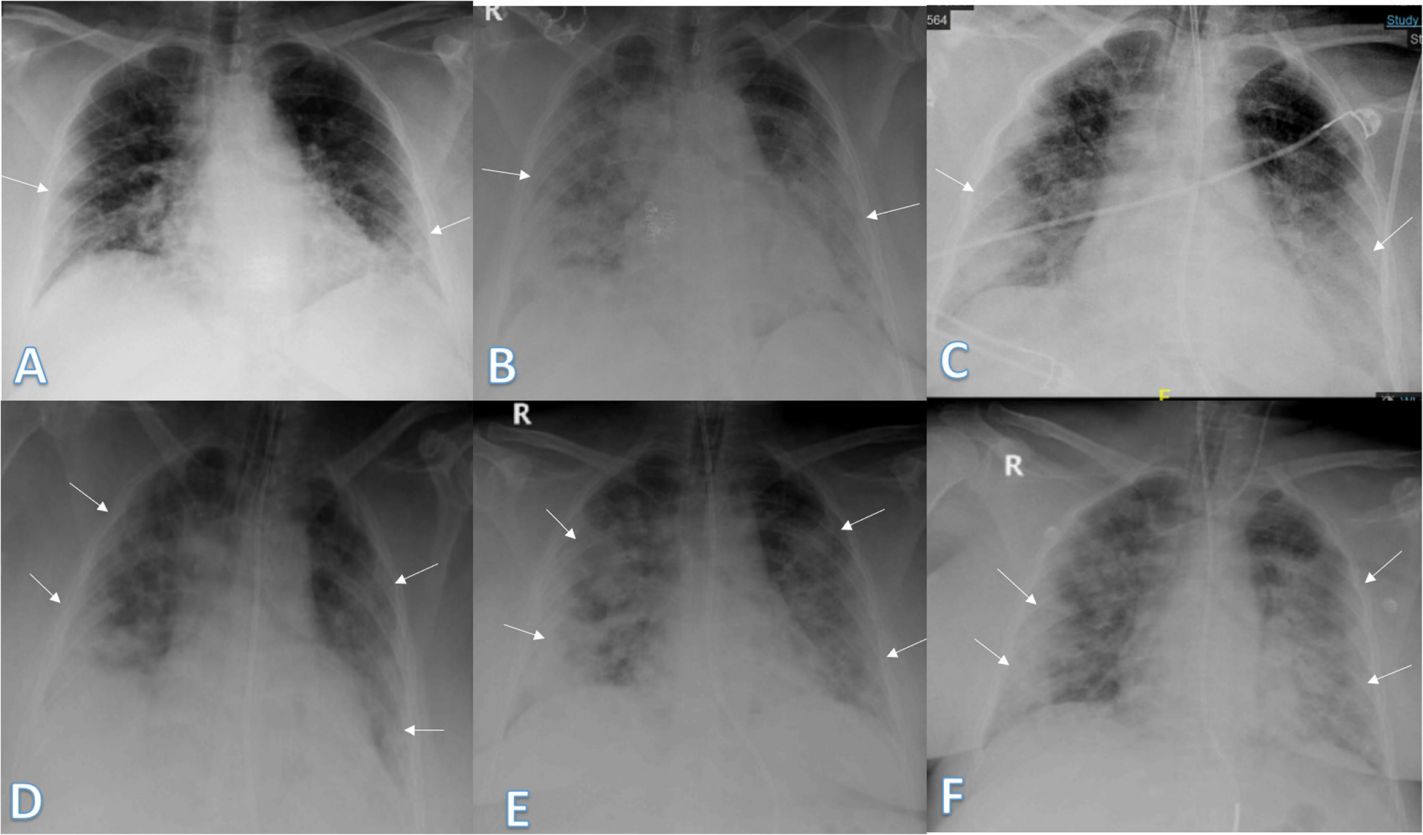
59-year female with positive COVID-19 virus. Initial X-ray (**a**) showed small peripheral right and left lower zonal air space consolidation opacities (arrows) with TSS as 2. On the 3rd day (**b**), air space consolidation opacities significantly increased extending to the upper and middle zones of both lungs (arrows) with TSS as 6 (3 on each side). The patient deteriorated and was intubated. On the 5th day (**c**), 7th day (**d**), 9th day (**e**), and 11th day (**f**), there was confluent bilateral air space consolidation opacities (arrows) with the severity score 4 on each side, so TSS was 8. The patient died on the 13th day

**Fig. 21:**
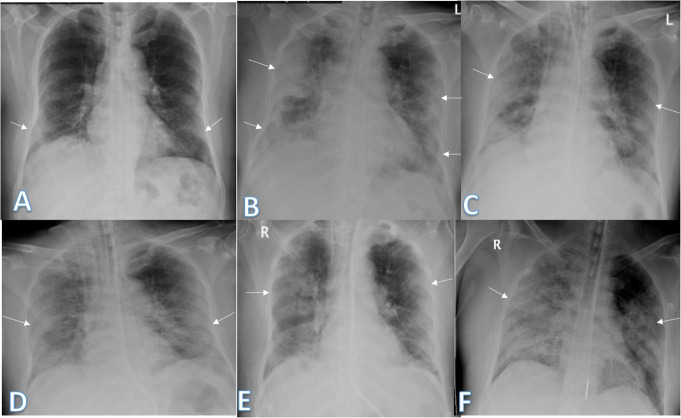
59-year male with positive COVID-19 virus. Initial X-ray (**a**) showed small peripheral right and left lower zonal air space consolidation opacities (arrows) with TSS as 2. On the 3rd day (**b**), air space consolidation opacities increased extended to the periphery of the upper and middle zones of both lungs (arrows) with TSS as 5 (3 on RT side and 2 on left side). The patient deteriorated and was intubated. On the 5th day (**c**), 6th day (**d**), 9th day (**e**) and 11th day (**f**), there was confluent consolidation (arrows) more on the right lung with severity score 4 on the right side and 3 on the left side, so TSS was 7. The patient died on the 11th day

In our study, the disease outcome showed a positive correlation with the maximum severity score (6.87 ± 0.71 for the dead patients and 2.06 ± 1.84 for the survived patients) with high statistical significance (*P* value < 0.001). In patients with TSS 2, there was a statistical significance between the TSS and the outcome of COVID disease for the survived patients (*P* value 0.032), while, in patients with TSS 7 and 8, there was a highly statistical significance for the outcome for the dead patients (*P* value < 0.001) (Figs. 20 and 21).

This agreed with Toussie et al. that showed that the severity of lung involvement on the initial chest radiograph was associated with more need for patients’ hospitalization as well as the increased risk of intubation and have proposed the use of initial CXR severity scores as a prognostic indicator of COVID-19 patients’ outcome.

The major strength of this study is the large sample size, which comprised of 350 COVID-19 patients. Our study had some limitations. First, it is a retrospective analysis. Second, the lack of correlation between CXR severity score and patient comorbidities (such as hypertension, diabetes, cardiovascular disease, and oncologic history). Third, not all the patients could be followed till the final outcome as the course of the disease was truncated in these patients. Fourth, CXR serial follow-up studies were not performed in a uniform pattern as it was dedicated by the clinician as regards the clinical condition. Fifth, for severe cases in the intensive care unit, the portable AP CXR was suboptimal with only few cases performed CT, so we could not judge the sensitivity of CXR.

## Conclusion

CXRs are a good monitor of COVID-19 chest manifestations and its scoring system provides an accurate method to predict the disease severity. Our study also revealed a positive correlation between the patients’ age and total severity score to the final disease outcome providing a good indicator for clinician to identify at an early stage the patients with the highest risk and plan specific treatment strategies for them.

## Data Availability

All data and material are available.

## Abbreviations

CXR: Chest X-ray
GGO: Ground glass opacities
TSS: Total severity score
WHO: World Health Organization
MERS: Middle Eastern respiratory syndrome
SARS: Severe acute respiratory syndrome
ICD: International Classification of Diseases
ARDS: Acute respiratory distress syndrome
CT: Computerized tomography
SD: Standard deviation
